# Effect of L-Tryptophan and L-Leucine on Gut Hormone Secretion, Appetite Feelings and Gastric Emptying Rates in Lean and Non-Diabetic Obese Participants: A Randomized, Double-Blind, Parallel-Group Trial

**DOI:** 10.1371/journal.pone.0166758

**Published:** 2016-11-22

**Authors:** Anne Christin Meyer-Gerspach, Simon Häfliger, Julian Meili, Alison Doody, Jens F Rehfeld, Jürgen Drewe, Christoph Beglinger, Bettina Wölnerhanssen

**Affiliations:** 1 Department of Biomedicine, University Hospital Basel, Basel, Switzerland; 2 Department of Research, St. Claraspital, Basel, Switzerland; 3 Diabetes Complications Research Centre, Conway Institute, School of Medicine, University College Dublin, Dublin, Ireland; 4 Department of Clinical Biochemistry, Rigshospitalet, University of Copenhagen, Copenhagen, Denmark; 5 Department of Clinical Pharmacology, University Hospital Basel, Basel, Switzerland; Garvan Institute of Medical Research, AUSTRALIA

## Abstract

**Background/Objectives:**

Gut hormones such as cholecystokinin (CCK) and glucagon-like peptide-1 (GLP-1) play a role as satiation factors. Strategies to enhance satiation peptide secretion could provide a therapeutic approach for obesity. Carbohydrates and lipids have been extensively investigated in relation to peptide release. In contrast, the role of proteins or amino acids is less clear. Our aim was to compare the effects of the amino acids L-tryptophan (L-trp) and L-leucine (L-leu) separately on gastric emptying and gut peptide secretion.

**Participants/Methods:**

The study was conducted as a randomized (balanced), double-blind, parallel-group trial. A total of 10 lean and 10 non-diabetic obese participants were included. Participants received intragastric loads of L-trp (0.52 g and 1.56 g) and L-leu (1.56 g), dissolved in 300 mL tap water; 75 g glucose and 300 mL tap water served as control treatments.

**Results:**

Results of the study are: i) L-trp at the higher dose stimulates CCK release (p = 0.0018), and induces a significant retardation in gastric emptying (p = 0.0033); ii) L-trp at the higher dose induced a small increase in GLP-1 secretion (p = 0.0257); iii) neither of the amino acids modulated subjective appetite feelings; and iv) the two amino acids did not alter insulin or glucose concentrations.

**Conclusions:**

L-trp is a luminal regulator of CCK release with effects on gastric emptying, an effect that could be mediated by CCK. L-trp’s effect on GLP-1 secretion is only minor. At the doses given, the two amino acids did not affect subjective appetite feelings.

**Trial Registration:**

ClinicalTrials.gov NCT02563847

## Introduction

Gut hormone secretion from enteroendocrine cells occurs in response to various nutrient components, including carbohydrates, fats and proteins. Carbohydrates, glucose in particular, and lipids, with a focus on long-chain fatty acids, have been extensively investigated in stimulation of incretin responses [1]. In contrast, the role of individual amino acids, the building blocks of proteins, in triggering gut hormone release has been less studied and remains an area of controversy. Gut hormones, such as cholecystokinin (CCK) and glucagon-like peptide-1 (GLP-1), play a role as satiation factors [2]. Strategies to enhance satiation peptide secretion could provide a therapeutic approach for treating obesity.

For example, an intraduodenal infusion of a mixture of tryptophan, valine, methionine and phenylalanine dose-dependently stimulated CCK release and bile salt secretion, whereas an intraduodenal administration of a mixture of arginine, histidine, leucine/isoleucine, lysine and threonine did not change CCK concentrations or bile salt secretion [3]. This finding supports the hypothesis that gut peptide release is dependent upon the nature of the specific nutrient. The aromatic amino acid, L-tryptophan, is of particular interest as previous studies have reported a potent effect on antropyloroduodenal motility and food intake [4, 5]. In addition, it has been shown that L-tryptophan stimulates CCK secretion when administered to humans [4, 6–8]. On the other hand, the branched-chain amino acid, L-leucine, can act as a nutrient signal to reduce food intake [9]. Whether supplementation of L-trp, in doses close to recommended daily amounts, and L-leu, in doses isocaloric to L-trp, affects CCK and GLP-1 release and appetite feelings warrants further investigation.

The primary aim of this present study was therefore to characterize the effect of L-tryptophan (L-trp) and L-leucine (L-leu) on the release of the satiation peptides, CCK and GLP-1, as well as on appetite sensations. A secondary outcome was to investigate gastric emptying rates of L-trp and L-leu. As the obese have a disturbed glycemic control with increased glucose and insulin concentrations [10], we were also interested in comparing the effects between lean and non-diabetic obese participants. The selection of the L-trp doses was based close to the daily intake recommended by the World Health Organization, WHO [11, 12]. For L-leu an isocaloric approach to L-trp was chosen.

## Materials and Methods

### Participants

A total of 10 lean volunteers (mean BMI: 21.7 ± 0.5 kg/m^2^, range 19.9–24.3 kg/m^2^; 5 men and 5 women; mean age: 24.6 ± 0.2 years, range 24–26 years), and 10 non-diabetic obese participants (mean BMI: 40.0 ± 1.4 kg/m^2^, range 33.8–48.2 kg/m^2^; 5 men and 5 women; mean age: 27.2 ± 2.8 years, range 20–48 years), took part in the study; all were healthy. The samples sizes for this study were chosen based on the results obtained in a pilot study using CCK release and gallbladder contraction to L-tryptophan as a parameter. L-tryptophan induced threshold to submaximal gallbladder contraction and a dose-dependent increase in CCK release. A sample size of 10 participants in each group will allow the detection of large differences in parameters (> 50%) between the treatment groups.

### Overall study design

The study was carried out in accordance with the Declaration of Helsinki; the protocol was submitted and approved by the Local Research and Ethics Committee in Basel (Ethikkommission Nordwest- und Zentralschweiz (EKNZ):2014–072; approval date: 16. May 2014). Participants were recruited by word of mouth (flyers on community notice boards) and followed-up over a five month period (20th May 2014—25th October 2014). Each participant provided written informed consent. The study is registered at ClinicalTrials.gov (NCT02563847) and occurred after enrolment of participants commenced. As this study protocol is part of a larger obesity cohort study, submitting this data would have entailed a sharing of confidential, sensitive information about our research plans. The authors confirm that all ongoing related trials for this intervention are registered. Exclusion from participation included smoking, substance abuse, regular intake of medications, psychiatric or medical illness (especially diabetes, exclusion criteria: fasting blood glucose ≥7.0 mmol/L) and any abnormalities detected on physical examination or laboratory screening. None of the participants had a history of gastrointestinal disorders, food allergies or dietary restrictions. Anthropometric measurements, including weight, height, BMI, as well as heart rate and blood pressure, were recorded. Participants were instructed to abstain from alcohol and strenuous exercise for 24 hours before each treatment. Participants consumed a simple carbohydrate-restricted standard dinner before 0800 PM and fasted from 1200 AM (midnight) onwards. Participants were admitted to the Phase 1 Research Unit of the University Hospital Basel at 0800 AM; an antecubital catheter was inserted into a forearm vein for blood collection.

Participants swallowed a radiopaque polyvinyl feeding tube (external diameter 8 French). The tube was placed through an anesthetized nostril; its intragastric position was confirmed by rapid injection of 10 mL of air and auscultation of the upper abdomen. The rationale for intragastric administration of the nutrients was to bypass oro-sensory cues and thus provide information on the isolated properties of nutrients, as well as on the relative role of the gastrointestinal tract in the secretion of satiation peptides and the short-term control of appetite. Volunteers were seated in a comfortable chair during the session.

### Experimental procedure

The study was conducted as a randomized (balanced, computer-based randomization), double-blind, placebo-controlled, parallel-group trial. Except for the intragastric infusions, the test trials were identical in design. Two doses (0.52 g = 2.48 mmoL and 1.56 g = 7.4 mmoL) of L-trp and an isocaloric dose of L-leu (1.56 g = 11.9 mmoL; isocaloric to 1.56 g L-trp) were selected. We used a 75 g load of glucose in 300 mL tap water as a positive control and 300 mL of pure tap water as a placebo treatment. Each test solution was labeled with 50 mg ^13^C-sodium acetate for determining gastric emptying. On 5 separate occasions, at least 3 days apart, participants received 1 of the 5 test solutions, in random order. The doses were well tolerated and did not induce any adverse effects. The intragastric infusions were freshly prepared each morning of the study and were at room temperature when administered. The study participant and study nurse (who carried out all tests), as well as the personnel performing analysis of blood samples, were blinded concerning the intragastric infusion administered.

After taking two fasting blood samples (t = -10 and -1 min) and a fasting breath sample (t = -1 min), participants received the test solution via the feeding tube within 2 minutes (t = 0–2 min). At regular time intervals (15, 30, 45, 60, 90 and 120 min), blood samples were taken into tubes containing EDTA (6 μmol/L), a protease inhibitor cocktail (cOmplete^™^, EDTA-free, 1 tablet/50 mL blood; Roche, Mannheim, Germany) and a dipeptidyl peptidase IV inhibitor (10 μL/mL; Millipore Corporation, St. Charles, Missouri, USA). Tubes, which had been placed on ice until processing, were centrifuged at 4°C at 3000 rpm for 10 min and plasma samples were processed into different aliquots. The total blood volume taken during one test day was 100 mL. All samples were stored at -70°C until analysis of plasma active GLP-1, CCK, insulin and glucose was performed.

The participant’s vital signs (blood pressure, heart rate) were measured before and after each study intervention.

### Assessment of appetite perceptions

Subjective appetite perceptions (feelings of hunger and prospective food consumption) were recorded immediately after each blood collection by using visual analog scales (VAS). The scales and scores have previously been designed and described [10, 13, 14].

### Assessment of gastric emptying

The gastric emptying rate was determined using a ^13^C-sodium acetate breath test, an accurate, non-invasive method for measuring gastric emptying, without radiation exposure, and a reliable alternative to scintigraphy, the current “gold standard” [15]. Test solutions were labeled with 50 mg of ^13^C-sodium acetate [15]. As described in detail previously [10], at fixed time intervals after administration of the test solution (15, 30, 45, 60, 75, 90, 105, 180, 210 and 240 min), end-expiratory breath samples were taken into a 100 mL foil bag. The ^13^C-exhalation was determined by non-dispersive infrared spectroscopy using an isotope ratio mass spectrophotometer (IRIS^®^; Wagner Analysen Technik, Bremen, Germany), and expressed as the relative difference (δ ‰) from the universal reference standard (carbon from Pee Dee Belemnite limestone). The calculation of percent of administered dose of ^13^C excreted per hour (%dose/h) has previously been described in detail [10].

### Materials

L-trp and L-leu (>97% pure) were purchased from Sigma Aldrich Chemical Company, Schnelldorf, Germany. Glucose was purchased from Hänseler, Herisau, Switzerland. ^13^C-sodium acetate was purchased from ReseaChem, Burgdorf, Switzerland.

### Laboratory analysis

#### Plasma active GLP-1

Plasma active GLP-1 was measured with a commercially available ELISA kit (Millipore Inc., St. Charles, Missouri, USA). The intra- and inter-assay variability is below 9.0% and 13.0%, respectively. The lowest GLP-1 concentration detected by this assay is 0.5 pmol/L in a 100 μL plasma sample.

#### Plasma CCK

Plasma CCK concentrations were measured with a sensitive radioimmunoassay using a specific antiserum (No. 92128) [16]. The intra- and inter-assay variability is below 15% for both. The lowest CCK concentration detected by this assay is 0.3 pmol/L in a 200 μL plasma sample.

#### Plasma insulin

Plasma insulin was measured with a commercially available electrochemiluminescence immunoassay (Cobas/Roche Diagnostics GmbH, Mannheim, Germany). The intra- and inter-assay coefficient of variation for this assay is below 2.0% and 2.8%, respectively. The lowest insulin concentration detected by this assay is 0.2 μU/mL in a 20 μL plasma sample.

#### Plasma glucose

Plasma glucose concentration was measured by a glucose oxidase method (Rothen Medizinische Laboratorien AG, Basel, Switzerland). The intra- and inter-assay coefficient of variation is below 2.9% and 3.9%, respectively. The lowest glucose concentration detected by this assay is 0.6 mmol/L in a 50 μL plasma sample.

After the 75 g glucose load, each Participant was classified, according to WHO criteria, as having impaired glucose tolerance (2 h blood glucose <11.1 mmol/L, but >7.8 mmol/L) or diabetes (fasting blood glucose ≥7.0 mmol/L and/or 2 h blood glucose ≥11.1 mmol/L) [17]. The 60 min blood glucose level after the 75 g glucose load was used as a predictor of the development of type 2 diabetes (with a risk factor limit of 8.6 mmol/L as risk factor) [18, 19].

### Statistical analysis

Descriptive statistics were used for demographic variables, such as age, weight, height, and BMI. Hormone and glucose profiles as well as VAS ratings were analyzed by calculating the change from baseline (the average of the two pre-infusion time points) for each post-infusion time point, and by calculating the area under the concentration-time curve from the delta values (iAUC, incremental AUC). Gastric emptying rates were analyzed by calculating the change from baseline (the single pre-infusion time point) for each post-infusion time point, and by calculating the area under the concentration-time curve from the delta values (iAUC). For gastric emptying we used iAUC0-60min, as gastric emptying of glucose occurs within 60min; all other parameters are presented as iAUC0-120min, reflecting the hormone profile. The parameters were tested for normality by the Shapiro-Wilk test method. Differences between the treatments were analyzed by generalized linear model repeated measures analysis of variance (ANOVA), using gender and BMI as cofactors. Post-hoc pairwise comparisons between the treatments were done using linear contrast with the Šidák correction. In case of significant deviation from normal distribution, treatments were compared using multiple Wilcoxon signed ranks tests with the Bonferroni-Holmes correction for multiplicity of testing.

Student’s unpaired t-test was used to test for significant differences between lean participants and non-diabetic obese participants in the different treatment groups (tap water, 75 g glucose, 0.52 g L-trp, 1.56 g L-trp, and 1.56 g L-leu).

All statistical analyses were performed using the statistical software package, SPSS for Windows, Version 24.0 (SPSS Inc., Chicago, Illinois, USA). Values were reported as mean ± SEM. Differences were considered to be significant when p<0.05.

## Results

**Fig 1:**
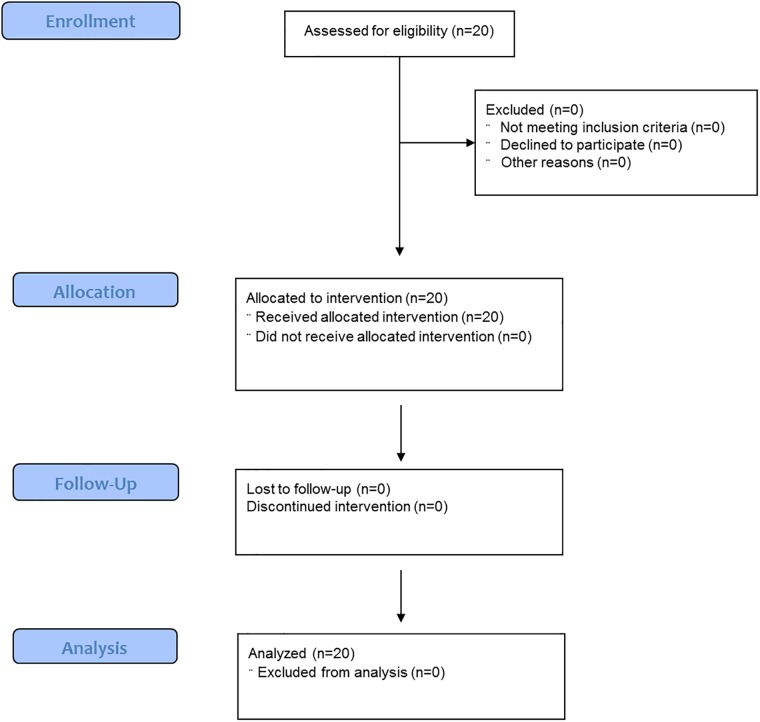
Flowchart. Adapted CONSORT flowchart for clinical trials.

All participants tolerated the study well and there were no adverse events. None of the participants had diabetes; three participants had impaired glucose tolerance and a risk factor for diabetes (according to the 60 min blood glucose levels after 75 g glucose). There were no drop-outs; complete data from 20 participants (10 lean and 10 non-diabetic obese) were available for analysis (Fig 1).

### Plasma CCK

**Fig 2:**
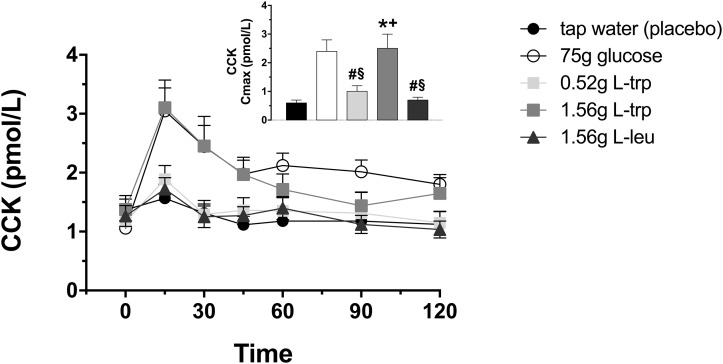
Plasma cholecystokinin (CCK). Plasma CCK concentrations in response to intragastric loads of 75 g glucose, 0.52 g L-trp, 1.56 g L-trp, and 1.56 g L-leu. Placebo treatment is 300 mL tap water. Data are expressed as mean ± SEM. * vs. tap water, p≤0.05; # vs. 75 g glucose, p≤0.05; § vs. 1.56 g L-trp; + vs. 0.52 L-trp. Statistical method: repeated measures ANOVA and simple contrasts with Šidák correction. n = 20 (10 men and 10 women). iAUC, incremental area under the concentration-time curve; CCK, cholecystokinin; L-trp, L-tryptophan; L-leu, L-leucine.

Infusion of L-trp induced an increase in CCK secretion with the higher dose, both in lean and non-diabetic obese participants (Fig 2, Table 1). The maximal plasma concentration (Cmax) in response to 1.56 g L-trp was statistically significant compared to tap water (p = 0.0018, Fig 2); the integrated increase was borderline statistically significant iAUC120: p = 0.0654; Table 1. The 1.56 g L-leu did not stimulate CCK secretion compared to tap water treatment (Fig 2, Table 1).

**Table 1:** Effect of L-trp and L-leu on plasma CCK, GLP-1, insulin and glucose concentrations as well as on gastric emptying rates and appetite feelings.

Hormones		tap water (A)	75 g Glucose (B)	0.52 g L-trp (C)	1.56 g L-trp (D)	1.56 g L-leu (E)
**CCK iAUC (0–120 min, pmol × min/L)**	lean	-3.4±17.1	153.2±43.9	15.0±14.9	77.7±18.3	7.7±16.6
	obese	-14.3±27.3	120.2±20.3	36.6±26.2	58.5±26.7	0.4±15.0
	total	-9.5±16.6	134.9±22.1	27.0±15.8	67.0±18.3	3.7±10.8
		p = 0.0001 (B)	p = 0.0001 (A)	p = 0.0021 (B)	p = 0.0654 (A)	p = 0.0002 (B)
		p = 0.0654 (D)	p = 0.0021 (C)			
			p = 0.0002 (E)			
**aGLP-1 iAUC (0–120 min, pmol × min/L)**	lean	45.6±60.8	714.5±105.4	113.9±54.2	225.6±95.9	54.9±51.2
	obese	47.2±27.9	502.6±92.0	100.8±42.7	251.2±81.6	4.3±58.2
	total	46.5±30.1	596.8±71.9	106.6±32.8	239.8±60.4	26.8±38.9
		p = 0.0002 (B)	p = 0.0002 (A)	p = 0.0001 (B)	p = 0.0153 (B)	p<0.0001 (B)
			p = 0.0001 (C)			
			p = 0.0153 (D)			
			p<0.0001 (E)			
**Insulin iAUC (0–120 min, μU × min/mL)**	lean	-159.2±175.5	3460.4±318.4	-40.2±23.2	-39.8±40.9	-115.2±34.5
	obese	-41.8±100.1	11411±2253	-14.9±126.8	199.6±111.4	304.4±176.4
	total	-13.2±5.0	7877.5±1558.7	-26.2±69.5	93.2±69.2	117.9±109.2
		p = 0.0004 (B)	p = 0.0004 (A,C,D)	p = 0.0004 (B)	p = 0.0004 (B)	p = 0.0006 (B)
			p = 0.0006 (E)			
**Glucose iAUC (0–120 min, mmol × min/L)**	lean	-3.6±7.6	121.7±44.1	-6.4±6.0	0.1±7.8	-17.3±4.6
	obese	-20.9±5.8	317.2±58.3	-16.8±8.5	-13.2±12.3	-13.5±3.1
	total	-13.2±5.0	230.3±43.7	-12.2±5.4	-7.3±7.6	-15.2±2.6
		p = 0.0003 (B)	p = 0.0003 (A)	p = 0.0004 (B)	p = 0.0007 (B)	p = 0.0001 (B)
			p = 0.0004 (C)			
			p = 0.0007 (D)			
			p = 0.0001 (E)			
**GE iAUC (0–60 min, % × min)**	lean	1388±91	761±56	1309±56	1140±83	1315±58
	obese	1433±83	914±57	1372±64	1328±67	1507±73
	total	1412±60	842± 43	1342±43	1239±43	1416±51
		p<0.0001 (B)	p<0.0001 (A,C-E)	p<0.0001 (B)	p = 0.0033 (A)	p<0.0001 (B)
		p = 0.0033 (D)			p<0.0001 (B)	p = 0.0248 (D)
					p = 0.0248 (E)	
**Hunger iAUC (0–120 min, cm × min)**	lean	55.5±110.5	-86.5±80.3	118.8±41.9	-13.2±82.9	55.8±52.0
	obese	-14.8±27.7	-27.9±48.5	5.1±66.4	20.9±43.8	-22.8±53.5
	total	18.5±53.3	-55.6±45.0	58.9±41.4	4.7±44.3	14.5±37.5
**P.f. consumption iAUC (0–120 min, cm × min)**	lean	-4.4±42.8	-77.0±80.6	36.1±50.8	-16.3±78.3	98.5±58.6
	obese	-30.2±42.8	-54.1±44.2	-1.1±84.4	-17.3±28.4	-16.3±55.4
	total	-18.0±52.8	-65.0±43.5	16.5±49.4	-16.8±38.8	38.1±41.4

GE, gastric emptying; iAUC, incremental area under the concentration-time curve. 0–60 min, time from start of test solution administration until 60 min after administration. 0–120 min, time from start of test solution administration until 120 min after administration. Data are expressed as mean ± SEM. (X), statistically significant difference *vs*. treatment X. Statistical method: repeated measures ANOVA and simple contrasts with Šidák correction. n = 10 lean (5 men and 5 women) and 10 non-diabetic obese (5 men and 5 women).

No significant difference was observed between 1.56 g L-trp and 75 g glucose administration. In contrast, 75 g glucose induced a significant increase in CCK concentrations compared to 0.52 g L-trp and 1.56 g L-leu (iAUC120: p = 0.0021 and p = 0.0002, respectively; Fig 2, Table 1).

Neither BMI nor gender significantly influenced CCK secretion.

### Plasma GLP-1

**Fig 3:**
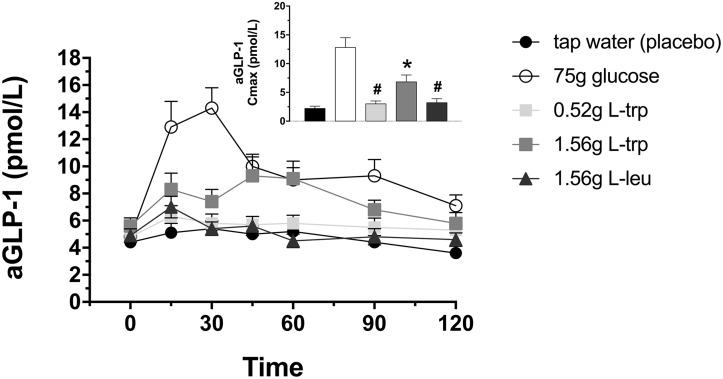
Plasma active glucagon-like peptide-1 (aGLP-1). Plasma aGLP-1 concentrations in response to intragastric loads of 75 g glucose, 0.52 g L-trp, 1.56 g L-trp, and 1.56 g L-leu. Placebo treatment is 300 mL tap water. Data are expressed as mean ± SEM. * vs. tap water, p≤0.05; # vs. 75 g glucose, p≤0.05; § vs. 1.56 g L-trp. Statistical method: repeated measures ANOVA and simple contrasts with Šidák correction. n = 20 (10 men and 10 women). aGLP-1, active glucagon-like peptide-1; iAUC, incremental area under the concentration-time curve; L-trp, L-tryptophan; L-leu, L-leucine.

Infusion of the higher dose L-trp induced an increase in aGLP-1 secretion (Fig 3). The maximal plasma concentration (Cmax) in response to 1.56 g L-trp was statistically significant compared to tap water (Cmax: p = 0.0257, Fig 3), however, the integrated effect did not reach statistical significance (Table 1). The 1.56 g L-leu did not significantly stimulate aGLP-1 secretion compared to tap water treatment (Fig 3, Table 1).

The 75 g glucose administration induced a significant increase in aGLP-1 concentrations compared to all amino acid treatments (iAUC120: p<0.02, respectively; Fig 3, Table 1).

No significant difference in the secretion of GLP-1 was found between lean and non-diabetic obese participants, or between males and females.

### Plasma insulin

BMI significantly influenced insulin concentrations in the fasting state (p = 0.006).

**Fig 4:**
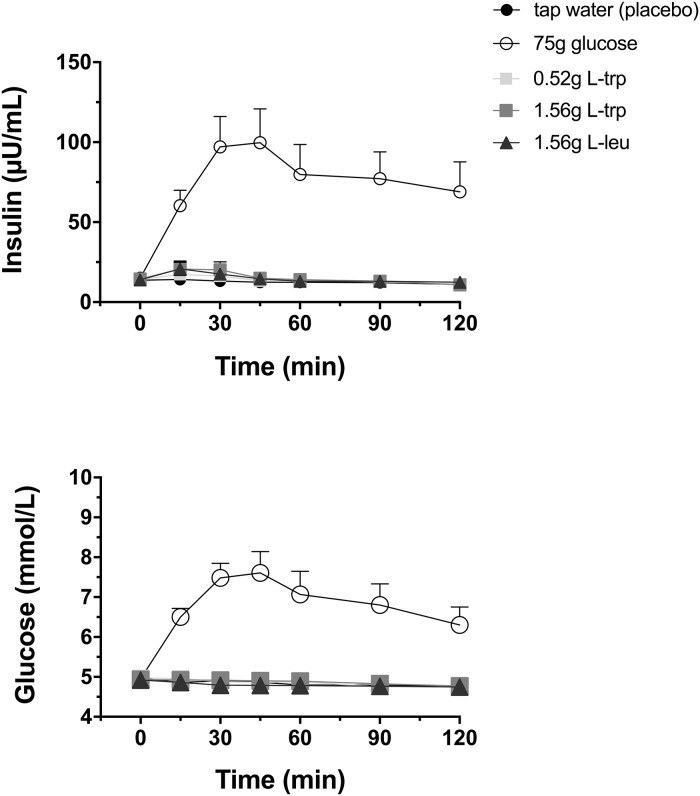
Plasma insulin and glucose. Plasma insulin and glucose concentrations in response to intragastric loads of 75 g glucose, 0.52 g L-trp, 1.56 g L-trp, and 1.56 g L-leu. Placebo treatment is 300 mL tap water. Data are expressed as mean ± SEM. Statistical method: repeated measures ANOVA and simple contrasts with with Šidák correction. n = 20 (10 men and 10 women).

Plasma insulin concentrations were not changed by the different amino acid infusions (Fig 4).

The 75 g glucose administration induced a significant increase in insulin concentrations compared to all amino acid treatments (iAUC120: p<0.001, respectively; Fig 4, Table 1).

Gender exerted no significant influence on insulin response.

### Plasma glucose

BMI significantly influenced glucose concentrations in the fasting state (p = 0.007). Plasma glucose concentrations were not changed by the different amino acid infusions (Fig 4, Table 1).

The 75 g glucose administration significantly increased plasma glucose concentrations compared to all amino acid treatments (iAUC120: p<0.001, respectively; Table 1).

Gender exerted no significant influence on glucose response.

### Gastric emptying rates

**Fig 5:**
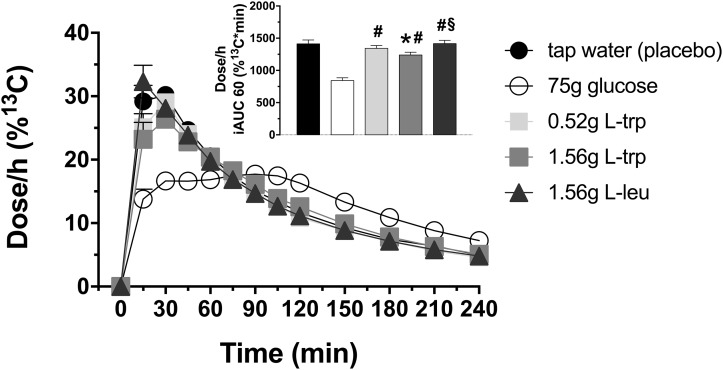
Gastric emptying rates. Gastric emptying rates in response to intragastric loads of 75 g glucose, 0.52 g L-trp, 1.56 g L-trp and 1.56 g L-leu. Placebo treatment is 300 mL tap water. Data are expressed as mean ± SEM. * vs. tap water, p≤0.05; # vs. 75 g glucose, p≤0.05; § vs. 1.56 g L-trp. Statistical method: repeated measures ANOVA and simple contrasts with Šidák correction. n = 20 (10 men and 10 women). iAUC, incremental area under the concentration-time curve; L-trp, L-tryptophan; L-leu, L-leucine.

After 1.56 g L-trp a significant retardation in gastric emptying was observed compared to tap water (iAUC60: p = 0.0033; Fig 5, Table 1). The 1.56 g L-leu had no effect (Fig 5 and Table 1).

The 75 g glucose administration did markedly slow gastric emptying compared to all amino acid treatments (iAUC60: p<0.0001, respectively; Fig 5, Table 1).

No significant differences in gastric emptying rates were observed between non-diabetic obese participants and lean controls after all treatments.

### Appetite perceptions

**Fig 6:**
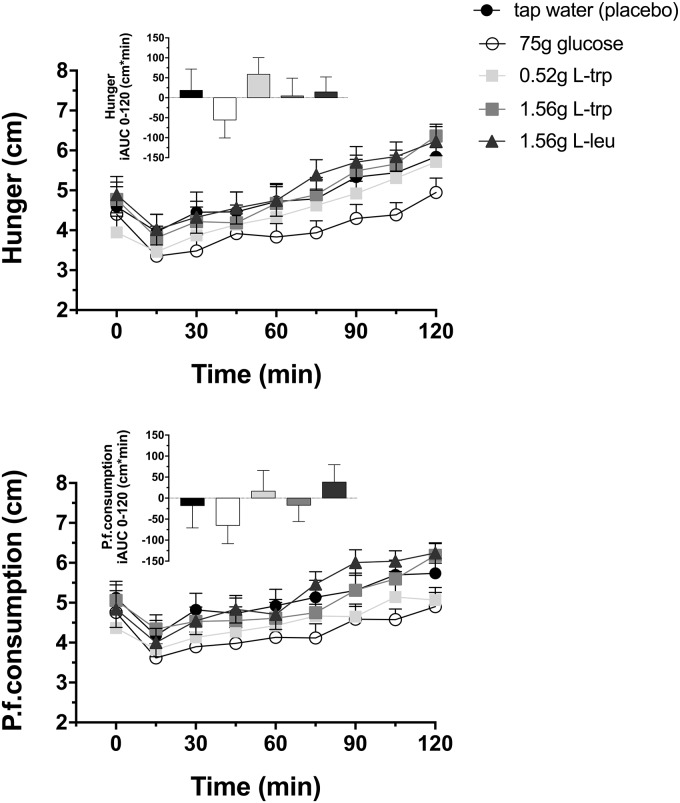
Feelings of hunger and prospective food consumption. Feelings of hunger and prospective food consumption in response to intragastric loads of 75 g glucose, 0.52 g L-trp, 1.56 g L-trp and 1.56 g L-leu. Placebo treatment is 300 mL tap water. Data are expressed as mean ± SEM. Statistical method: repeated measures ANOVA and simple contrasts with Šidák correction. n = 20 (10 men and 10 women). P.f.consumption, prospective food consumption; iAUC, incremental area under the concentration-time curve; L-trp, L-tryptophan; L-leu, L-leucine.

Feelings of hunger and prospective food consumption were not changed by the different amino acid infusions compared to tap water (Fig 6, Table 1).

The 75 g glucose administration induced a numerical reduction in subjective feelings of hunger and prospective food consumption compared to all amino acid treatments; the effect did, however, not reach the level of statistical significance (Fig 6, Table 1).

## Discussion

Luminal enteral communication is a key factor in the regulation of appetite, food intake and metabolism. Important mediators of these effects include the gut hormones, CCK and GLP-1. [2] Carbohydrates, proteins and fat can stimulate both hormones. Previous studies have highlighted the importance of fat digestion in the regulation of CCK and GLP-1 release: Only free fatty acids with chain lengths >12 carbons are able to stimulate both hormones. This is consistent with the physical properties of these molecules as free fatty acids with >12 carbons and, as such, solid lipids that require emulsification by bile salts for digestion [20]. The effect of protein digestion on the release of these hormones is less clear. Protein, however, exerts potent effects on appetite and modulates glycemic responses to food ingestion [21, 22]. With this background in mind, we investigated the effects of two amino acids, L-trp and L-leu separately, on CCK and GLP-1 secretions, along with their effect on gastric emptying and feelings of hunger and prospective food consumption, both in lean and non-diabetic obese participants. Other studies have shown that both amino acids modulate appetite and/or glucose metabolism [4, 23, 24]. In this trial, we supplied the two amino acids and based the selection of the L-trp doses close to the daily intake recommended by the WHO [11, 12]; for L-leu an isocaloric approach to the 1.56 g L-trp dose was chosen.

The results of the study can be summarized as follows: i) L-trp, at the higher dose, stimulates CCK release (>4 fold increase compared to tap water, p = 0.0018), and induced a significant retardation in gastric emptying (p = 0.0033); ii) L-trp, at the higher dose, induced a small increase in GLP-1 secretion (3 fold increase compared to tap water, p = 0.0257); iii) none of the amino acids modulated subjective appetite feelings; iv) the two amino acids did not change insulin or glucose concentrations.

In light of *in vitro* studies demonstrating that the individual L-amino acids, phenylalanine, leucine, glutamate and tryptophan, provoke CCK secretion (but not their D-isoforms) [25], we selected L-trp and L-leu for this study. Early studies from the end of the last century provided experimental evidence that, through the release of CCK, L-trp modulates various digestive functions, including bile salt and pancreatic enzyme secretion, as well as appetite [3, 7, 26]. Along these lines, it has been proposed that CCK plays a role in the regulation of GLP-1 release which, in turn, could modulate digestive and metabolic processes [20]. Here we document that low doses of L-trp, in the range of recommended daily intake, stimulate CCK release (> 4 fold increase compared to tap water) and have a small effect on GLP-1 (3 fold increase compared to tap water) secretion, whereas an isocaloric load of L-leu affected neither CCK nor GLP-1 release. The latter corresponds to a recent study by Steinert *et al*. [9]: Intraduodenal administration of L-leu exerted no effect on plasma GLP-1 or PYY levels. The effect of L-trp on CCK and GLP-1 release in the current study was only small compared to the effects of glucose administration, but confirm and extend the role of luminal L-trp as one of the triggers stimulating a cascade of digestive functions.

L-trp had a significant effect on gastric emptying; the retardation of gastric emptying is most likely mediated by CCK, although final experimental proof is lacking. Unfortunately, specific CCKA receptor antagonists, such as dexloxiglumide or devazepide, are no longer available for human use [27, 28].

We, and others, have previously reported that non-diabetic obese adolescents and adults have an attenuated GLP-1 response to meal ingestion compared to lean persons [29–31]. The mechanism of the attenuated response is not entirely clear. Here, no difference in GLP-1 secretion was observed between lean and non-diabetic obese participants.

An objective of this study was to investigate new strategies for enhancing satiation peptide secretion in order to reduce appetite, and potentially food intake, by supplementing the amino acids at doses close to recommendations (WHO). These results clearly indicate that the recommended doses are insufficient to achieve these effects.

Some potential limitations of the present study require consideration. First, the aim of the study was to investigate the effect of L-trp and L-leu on gastric emptying and gut peptide secretion (doses for L-trp were close to recommended daily intake); we cannot exclude that higher, pharmacological doses of L-trp and L-leu or a combination of different specific amino acids, would have more potent effects on the secretion of gut peptides and appetite feelings. Second, we report effects of L-trp and L-leu only on fasting blood glucose. Determination of the effects of the two amino acids on postprandial glycemia requires concomitant administration of carbohydrates. Third, we used an intragastric infusion paradigm, a frequently used and well-tolerated procedure. The rational for intragastric administration of the nutrients was to bypass oro-sensory cues and thus provide information on the isolated properties of nutrients, and the relative role of the gastrointestinal tract in the secretion of satiation peptides and the short-term control of appetite. However, this may potentially interfere with normal physiological function; we can only speculate to what extent the observed effects may reflect those when food is ingested orally. Fourth, it should be recognized that, under physiological conditions (when protein is ingested orally), approximately two-thirds of protein absorption occurs as di- and tripeptides (not free amino acids), in the upper small intestine; therefore, the effect of di-and tripeptides on gut function and satiation warrants investigation. Fifth, the variance of some parameters was higher than anticipated; our power calculations were based on a pilot study using the difference in CCK release in response to both amino acids as response parameter; based on these calculations 10 subjects per group would be sufficient to detect the expected differences in the measured outcome parameters; unfortunately this was not the case for appetite perceptions. Finally, we only studied lean and non-diabetic obese participants; effects in T2DM remain to be established.

In conclusion, L-trp participates in the regulation of CCK release and affects gastric emptying rates; in contrast, L-leu at the dose given exerts no digestive effects. The role of L-trp on GLP-1 release was minor. Finally, the administration of the two amino acids did not change glucose concentrations in either lean or in non-diabetic obese participants.

## Supporting Information

S1 CONSORT Checklist (PDF)

S1 File (PDF)

## References

[1] Diakogiannaki E, Gribble FM, Reimann F: Nutrient detection by incretin hormone secreting cells. *Physiol Behav* 2012, 106:387–393. 10.1016/j.physbeh.2011.12.001 22182802 PMC3361765

[2] Cummings DE, Overduin J: Gastrointestinal regulation of food intake. *J Clin Invest* 2007, 117:13–23. 10.1172/JCI30227 17200702 PMC1716217

[3] Colombel JF, Sutton A, Chayvialle JA, Modigliani R: Cholecystokinin release and biliopancreatic secretion in response to selective perfusion of the duodenal loop with aminoacids in man. *Gut* 1988, 29:1158–1166. 3197988 10.1136/gut.29.9.1158 PMC1434373

[4] Steinert RE, Luscombe-Marsh ND, Little TJ, Standfield S, Otto B, Horowitz M, et al: Effects of intraduodenal infusion of L-tryptophan on ad libitum eating, antropyloroduodenal motility, glycemia, insulinemia, and gut peptide secretion in healthy men. *J Clin Endocrinol Metab* 2014, 99:3275–3284. 10.1210/jc.2014-1943 24926954

[5] Carney BI, Jones KL, Horowitz M, Sun WM, Hebbard G, Edelbroek MA: Stereospecific effects of tryptophan on gastric emptying and hunger in humans. *J Gastroenterol Hepatol* 1994, 9:557–563. 7865713 10.1111/j.1440-1746.1994.tb01560.x

[6] Liddle RA: Regulation of cholecystokinin secretion in humans. *J Gastroenterol* 2000, 35:181–187. 10755686 10.1007/s005350050328

[7] Ballinger AB, Clark ML: L-phenylalanine releases cholecystokinin (CCK) and is associated with reduced food intake in humans: evidence for a physiological role of CCK in control of eating. *Metabolism* 1994, 43:735–738. 8201963 10.1016/0026-0495(94)90123-6

[8] Thomas FB, Sinar D, Mazzaferri EL, Cataland S, Mekhjian HS, Caldwell JH, et al: Selective release of gastric inhibitory polypeptide by intraduodenal amino acid perfusion in man. *Gastroenterology* 1978, 74:1261–1265. 648819

[9] Steinert RE, Landrock MF, Ullrich SS, Standfield S, Otto B, Horowitz M, et al: Effects of intraduodenal infusion of the branched-chain amino acid leucine on ad libitum eating, gut motor and hormone functions, and glycemia in healthy men. *Am J Clin Nutr* 2015, 102:820–827. 10.3945/ajcn.115.114488 26289436

[10] Meyer-Gerspach AC, Cajacob L, Riva D, Herzog R, Drewe J, Beglinger C, et al: Mechanisms Regulating Insulin Response to Intragastric Glucose in Lean and Non-Diabetic Obese Subjects: A Randomized, Double-Blind, Parallel-Group Trial. *PLoS One* 2016, 11:e0150803 10.1371/journal.pone.0150803 26942445 PMC4778796

[11] Moehn S, Pencharz PB, Ball RO: Lessons learned regarding symptoms of tryptophan deficiency and excess from animal requirement studies. *J Nutr* 2012, 142:2231S–2235S. 10.3945/jn.112.159061 23077198

[12] Joint WHOFAOUNUEC: Protein and amino acid requirements in human nutrition. *World Health Organ Tech Rep Ser* 2007:1–265, back cover. 18330140

[13] Blundell J, de Graaf C, Hulshof T, Jebb S, Livingstone B, Lluch A, Mela D, et al: Appetite control: methodological aspects of the evaluation of foods. *Obes Rev* 2010, 11:251–270. 10.1111/j.1467-789X.2010.00714.x 20122136 PMC3609405

[14] Flint A, Raben A, Blundell JE, Astrup A: Reproducibility, power and validity of visual analogue scales in assessment of appetite sensations in single test meal studies. *Int J Obes Relat Metab Disord* 2000, 24:38–48. 10702749 10.1038/sj.ijo.0801083

[15] Ghoos YF, Maes BD, Geypens BJ, Mys G, Hiele MI, Rutgeerts PJ, et al: Measurement of gastric emptying rate of solids by means of a carbon-labeled octanoic acid breath test. *Gastroenterology* 1993, 104:1640–1647. 8500721 10.1016/0016-5085(93)90640-x

[16] Rehfeld JF: Accurate measurement of cholecystokinin in plasma. *Clin Chem* 1998, 44:991–1001. 9590372

[17] Alberti KG, Zimmet PZ: Definition, diagnosis and classification of diabetes mellitus and its complications. Part 1: diagnosis and classification of diabetes mellitus provisional report of a WHO consultation. *Diabet Med* 1998, 15:539–553. 9686693 10.1002/(SICI)1096-9136(199807)15:7<539::AID-DIA668>3.0.CO;2-S

[18] Abdul-Ghani MA, Williams K, DeFronzo RA, Stern M: What is the best predictor of future type 2 diabetes? *Diabetes Care* 2007, 30:1544–1548. 10.2337/dc06-1331 17384342

[19] Trahair LG, Horowitz M, Marathe CS, Lange K, Standfield S, Rayner CK, et al: Impact of gastric emptying to the glycemic and insulinemic responses to a 75-g oral glucose load in older subjects with normal and impaired glucose tolerance. *Physiol Rep* 2014, 2. 10.14814/phy2.12204 PMC4255811 25413324

[20] Beglinger S, Drewe J, Schirra J, Goke B, D'Amato M, Beglinger C: Role of fat hydrolysis in regulating glucagon-like Peptide-1 secretion. *J Clin Endocrinol Metab* 2010, 95:879–886. 10.1210/jc.2009-1062 19837920

[21] Bowen J, Noakes M, Clifton PM: Appetite regulatory hormone responses to various dietary proteins differ by body mass index status despite similar reductions in ad libitum energy intake. *J Clin Endocrinol Metab* 2006, 91:2913–2919. 10.1210/jc.2006-0609 16735482

[22] Belza A, Ritz C, Sorensen MQ, Holst JJ, Rehfeld JF, Astrup A: Contribution of gastroenteropancreatic appetite hormones to protein-induced satiety. *Am J Clin Nutr* 2013, 97:980–989. 10.3945/ajcn.112.047563 23466396

[23] Kalogeropoulou D, Lafave L, Schweim K, Gannon MC, Nuttall FQ: Leucine, when ingested with glucose, synergistically stimulates insulin secretion and lowers blood glucose. *Metabolism* 2008, 57:1747–1752. 10.1016/j.metabol.2008.09.001 19013300

[24] Layman DK, Walker DA: Potential importance of leucine in treatment of obesity and the metabolic syndrome. *J Nutr* 2006, 136:319S–323S. 16365106 10.1093/jn/136.1.319S

[25] Daly K, Al-Rammahi M, Moran A, Marcello M, Ninomiya Y, Shirazi-Beechey SP: Sensing of amino acids by the gut-expressed taste receptor T1R1-T1R3 stimulates CCK secretion. *Am J Physiol Gastrointest Liver Physiol* 2013, 304:G271–282. 10.1152/ajpgi.00074.2012 23203156 PMC3566511

[26] Dooley CP, Valenzuela JE: Duodenal volume and osmoreceptors in the stimulation of human pancreatic secretion. *Gastroenterology* 1984, 86:23–27. 6689670

[27] Liddle RA, Gertz BJ, Kanayama S, Beccaria L, Coker LD, Turnbull TA, et al: Effects of a novel cholecystokinin (CCK) receptor antagonist, MK-329, on gallbladder contraction and gastric emptying in humans. Implications for the physiology of CCK. *J Clin Invest* 1989, 84:1220–1225. 10.1172/JCI114288 2794058 PMC329781

[28] Meyer BM, Werth BA, Beglinger C, Hildebrand P, Jansen JB, Zach D, et al: Role of cholecystokinin in regulation of gastrointestinal motor functions. *Lancet* 1989, 2:12–15. 2567793 10.1016/s0140-6736(89)90255-9

[29] Verdich C, Toubro S, Buemann B, Lysgard Madsen J, Juul Holst J, Astrup A: The role of postprandial releases of insulin and incretin hormones in meal-induced satiety—effect of obesity and weight reduction. *Int J Obes Relat Metab Disord* 2001, 25:1206–1214. 10.1038/sj.ijo.0801655 11477506

[30] Meyer-Gerspach AC, Wolnerhanssen B, Beglinger B, Nessenius F, Napitupulu M, Schulte FH, et al: Gastric and intestinal satiation in obese and normal weight healthy people. *Physiol Behav* 2014, 129:265–271. 10.1016/j.physbeh.2014.02.043 24582673

[31] Beglinger S, Meyer-Gerspach AC, Graf S, Zumsteg U, Drewe J, Beglinger C, et al: Effect of a test meal on meal responses of satiation hormones and their association to insulin resistance in obese adolescents. *Obesity (Silver Spring)* 2014, 22:2047–2052. 24930697 10.1002/oby.20805

