# Supplementary material for: Effect of L-Tryptophan and L-Leucine on Gut Hormone Secretion, Appetite Feelings and Gastric Emptying Rates in Lean and Non-Diabetic Obese Participants: A Randomized, Double-Blind, Parallel-Group Trial
Source: PLoS One. 2016 Nov 22;11(11):e0166758. doi: 10.1371/journal.pone.0166758 (PMC5119776; doi:10.1371/journal.pone.0166758)
Supplement: S1 File — (PDF) [file pone.0166758.s002.pdf]

Universitätsspital Basel

Gastroenterologie und Hepatologie

Effect of amino acids on gastric emptying and release of satiation peptides in normal weight and obese subjects

Version 2:

Principal Investigator: **Dr. Bettina Wölnerhanssen (MD)**
Division of Gastroenterology and Hepatology/
Clinical Research
University Hospital Basel

Signature: _________________

Investigator: **Dr. Anne Christin Meyer-Gerspach (PhD)**
Division of Gastroenterology and Hepatology/
Clinical Research
University Hospital Basel

Signature: _________________

Sponsor: **Prof. Dr. Christoph Beglinger**

Division of Gastroenterology and Hepatology
University Hospital Basel

Signature: ________________

Contents

GENERAL CONSIDERATIONS

Ethical standards	3
Informed consent	3
Registration	3
Data protection	3
Withdrawal from the study	3
List of co-workers	4

SCIENTIFIC PART

Introduction	4
Hypotheses and Aims	5
Methods	5
Overall design	5
Study subjects	6
Experimental procedure	7
Measurements and analysis	8
References	11

GENERAL CONSIDERATIONS

Ethical Standards and Study site

All studies will be performed in accordance with the Declaration of Helsinki, International Conference on Harmonization, Guidelines for Good Clinical Practice (GCP) and standard operating procedures of the participating centers. Trained study personnel will perform any research related procedures such as obtaining venous access and collect blood samples. Trained study personnel will be present during all research related sessions, while also be supported by study physicians. Studies will be carried out in the University Hospital of Basel (Sponsor: Prof. Dr. Ch. Beglinger, Investigators: Dres. Anne Christin Meyer-Gerspach and Bettina Wölnerhanssen). Sponsor and Investigators all obtain a GCP-certificate and have vast experience in carrying out similar studies.
The investigators will provide periodic reports as required by the Ethics Committee. For this trial a formal monitoring is not planned.

Informed consent

All subjects will give written informed consent before any participation in the study. The investigator obtaining consent will also sign and date the consent form. All studies will be approved by the respective Ethics Committees. Participation is voluntary. Participants will not receive any financial incentives to participate in the study.

Registration

The study will be registered at clinicaltrials.gov.

Data protection

All data will be anonymized and all information will be treated in strict confidence. Participants have the right to gain access to their results and the Ethics Committee is at liberty to undertake inspections and gain access to original data. All data is archived for 10 years. Blood samples are stored for 5 years and will be destroyed.

Withdrawal from the study

Should participants require withdrawal for any reason at any time patients will be offered a final medical examination. In case of withdrawal, only data/samples collected during the time period prior to withdrawal will be used for the study.

List of co-workers:

- Prof. Ch. Beglinger, Gastroenterology University Hospital Basel.
  Function: Sponsor
- Dr. phil. A. Meyer-Gerspach, Clinical Research, Gastroenterology University Hospital Basel.
  Function: Investigator
- Dr. med. B Wölnerhanssen, Clinical Research, Gastroenterology University Hospital Basel.
  Function: Investigator

SCIENTIFIC PART

Introduction

Protein is currently believed to exert the greatest appetite-suppressing effect of the three macronutrients (carbohydrates, fats and proteins) in animals and humans (1). High-protein diets have been extensively studied for their ability to reduce total energy intake and body weight (1). Mechanisms that have been suggested include stimulation of insulin release (2), postprandial thermogenesis (3), intestinal gluconeogenesis (4), and direct effects of amino acids in regions of the brain (5). In addition, it has been hypothesized that protein-induced satiation could be due to alterations in the release of gastrointestinal satiation peptides, such as cholecystokinin (CCK), glucagon-like peptide-1 (GLP-1) and peptide tyrosine tyrosine (PYY) (5). Administration of different high-protein diets induced prolonged or higher concentrations of CCK, GLP-1 and PYY compared to normal-protein diets in normal or overweight patients (2, 6-9). In addition, there is evidence that, like fat, protein digestion to dipeptides or tripeptides and free amino acids enhances gastrointestinal hormone release (10). Already in 1956, it was suggested that an elevated concentration of plasma amino acids serve as a satiation signal for food intake and thereby results in depressed food intake (11). To date the effect of amino acids on satiation peptide release is only rarely studied. Gannon *et al.* (12) studied the effect of different orally administered amino acids on plasma insulin, glucose and glucagon levels. The majority of amino acids did not significantly affect the glucose levels, however, induced an increase in both, circulating insulin and glucagon

(12). These results were in line with a number of previous data showing increased insulin and glucagon release after administration of different amino acids (reviewed in (12)). GLP-1 is a well-known incretin hormone and it remains established whether the amino acid induced increase in insulin is mediated via increased secretion of GLP-1. Greenfield *et al.* (13) studied the effect of glutamine on GLP-1, glucagon and insulin concentrations in human subjects. They found that glutamine increases circulating GLP-1, insulin and glucagon in lean, obese and type 2 diabetes subjects (13).

In our own pilot project we found, that the specific amino acid L-tryptophan is a potent stimulus of gallbladder contraction and that this effect is probably mediated through the dose-dependent increase in CCK levels. These results are in line with previous observations showing that L-tryptophan and L-phenylalanine stimulate CCK release in healthy humans (14-17). A possible mechanism involved includes amino acids induced activation of CaR, which expression was found in CCK releasing I-cells of the gut (18, 19). Details on specific amino acid stimulated secretion of other satiation peptides, such as GLP-1 and PYY, in humans are, to date, not available.

Hypotheses

Specific amino acids in the GI tract stimulate the release of satiation factors (GLP-1 and PYY) which is probably mediated through mechanisms involving the activation of the CaR and subsequent release of CCK.

Aim

To investigate the effect of intragastric (IG) amino acids on satiation peptide release and gastric emptying and appetite perception.

Methods

Overall design

The study will be performed as a randomized, double-blind, placebo-controlled, crossover study. A randomization list is prepared in advance by computer-randomization (Excel). Double-blind: the investigator (Dr. Anne Christin Meyer-Gerspach) who will administer the solution and the respective study subject are blinded as to the content of the solution. Only the principal investigator (Dr. B. Wölnerhanssen) has access to the randomization list and will prepare the solutions according to it in advance.

Each subject will be studied on 5 occasions with at least three days apart. Sessions will take about 5 hours. Subject's food intake on the preceding day of each study day will be standardized: they will consume an identical meal before 8 p.m. and fast from 10 p.m. In addition, subjects will refrain from alcohol and strenuous exercise for 24 hours before each treatment. Subjects will be admitted to the Phase 1 Research Unit at 8:00 a.m. on each study day. The treatments of each part will be identical in design except for the intragastric perfusion.

Study subjects

Ten healthy normal weight and ten healthy obese volunteers will be recruited. Recruitment is achieved by word-of-mouth. The screening procedure will include the following assessments: a medical interview and a full physical examination. Anthropometric measurements including weight, height, BMI, as well as heart rate and blood pressure will be recorded for all participants. In women of child bearing age, a urine pregnancy test will be carried out. There is no safety risk in this study for pregnant women; however, pregnancy might influence results.

The following inclusion criteria are applied:

- Healthy normal weight subjects with a body-mass index of 19.0-24.9
- Healthy obese subjects with a body-mass index of > 30
- Normal eating habits (no diets; no dietary changes; no special dietary habits, such as vegetarian/vegan)
- Age 18-40 years
- Stable body weight for at least three months

Exclusion criteria:

- Smoking
- Substance abuse
- Regular intake of medications (except for oral contraceptives)
- Medical or psychiatric illness
- History of gastrointestinal disorders
- Food allergies
- Pregnancy, breast feeding

The amendment will be submitted and approved by the Human Ethical Research Committee of the University of Basel. All subjects will have to give written informed consent.

Experimental procedure

Effect of intragastric (IG) infusion of amino acids on satiation peptide release, gastric emptying rates and appetite perception

Subjects will swallow a feeding tube. The IG position of the feeding tube will be verified by rapid injection of 10 mL of air and auscultation of the upper abdomen. In addition, an antecubital vein catheter will be inserted for blood drawing. After taking two fasting blood samples (-15, -1 min), the test solutions will be infused intragastrically within 2 minutes. The feeding tube will be removed immediately after the infusion is completed. Test solutions compounds are listed in table 1. The order of treatments will be randomized.

At regular time intervals (15, 30, 45, 60, 90, 120 and 180 min) blood samples will be collected on ice into tubes containing EDTA (6 µmol/L), a protease inhibitor cocktail (Complete®, EDTA-free, 1 tablet/50 mL blood; Roche, Mannheim, Germany) and a dipeptidylpeptidase IV inhibitor (10 µL/mL; Millipore Corporation, St. Charles, Missouri, USA). Tubes will be centrifuged at 4 C° at 3 000 rpm for 10 min and plasma samples will be processed into different aliquots. All samples will be stored at -70 C° until analysis of metabolites including glucose and amino acid profiles as well as of hormonal responses including insulin, GLP-1, CCK, PYY and ghrelin. The total blood volume taken during one test day will be about 100 mL. Immediately after each blood collection, appetite perceptions, such as feelings of hunger, prospective food consumption, fullness and satiety will be recorded using visual analogue scales (VAS). For determination of the gastric emptying rates, end-expiratory breath samples will be taken at fixed time intervals after instillation of the test solution.

Table 1: Test solutions compounds for the different treatments (visits)

	**Test solutions compounds**
**Visit A**	1.56 g (7.5 mmol) L-tryptophan + 50 mg ^{13}C-Sodium Acetate in 300 mL tap water
**Visit B**	0.52 g (2.48 mmol) L-tryptophan + 50 mg ^{13}C-Sodium Acetate in 300 mL tap water
**Visit C**	1.56 g (11.89 mmol) L-leucine + 50 mg ^{13}C-Sodium Acetate in 300 mL tap water
**Visit D**	75 g glucose + 50 mg ^{13}C-Sodium Acetate in 300 mL tap water
**Visit E**	300 mL tap water + 50 mg ^{13}C-Sodium Acetate

Materials

L-tryptophan and L-leucine will be purchased from Sigma Aldrich Chemical Company, Germany (>97% pure). Glucose will be purchased from Hänseler, Switzerland and ^{13}C-sodium acetate from ReseaChem, Switzerland.

Amino acids solutions will be administered in physiological amounts according to our pilot project. The IG infusions will be freshly prepared each morning of the study and will be at room temperature when administered.

Measurements and analysis

Assessment of appetite

Appetite perceptions (feelings of hunger, fullness, satiation and prospective food consumption) will be scored at regular intervals using visual analogue scales (VAS). These scores are based on validated data described by Blundell and have been used extensively by other investigators, but also by our own group (20-24). VAS consists of a horizontal, unstructured, 100 mm line with words anchored at each end describing the extremes of a unipolar question (most positive and most negative rating). Subjects assign a vertical mark across the line to index the magnitude of their subjective sensation. To ensure reliable and valid results, subjects cannot refer to their previous ratings. In addition, they are instructed to take enough time to rate their appetite sensation as precisely as possible. Quantifications of the measurements are made by measuring the distance from the left end of the line to the mark. The scales and scores have previously been designed and described in more detail (25).

Assessment of gastric emptying rates

Gastric emptying rates will be assessed using the ^{13}C-sodium acetate breath test. This test is an accurate, non-invasive, simple method without radiation exposure, and represents a reliable alternative to scintigraphy, the gold standard for measuring gastric emptying (26, 27). The test solutions will be labelled with 50 mg ^{13}C-sodium acetate; the substrate is rapidly absorbed in the proximal small intestine, metabolized in the liver with the production of $^{13}CO_2$ which is exhaled rapidly, thus, reflecting gastric emptying of nutrients (26, 27). Subjects will be asked to exhale through a mouth-piece to collect an end-expiratory breath sample into a foil bag at certain time intervals. The $^{13}CO_2$ breath

content will be determined by non-dispersive infrared spectroscopy using an isotope ratio mass spectrophotometer (IRIS; Wagner Analysen Technik, Bremen, Germany). ^{13}C-abundance in breath is expressed as relative difference (δ ‰) from the universal reference standard (carbon from Pee Dee Belemnite limestone). ^{13}C-enrichment is defined as the difference between preprandial ^{13}C-abundance in breath and ^{13}C-abundance at the defined time points postprandially and is given in δ over basal (DOB, ‰). Based on these values, time to reach maximal emptying speed and areas under the curve of the responses will be calculated (27, 28).

Laboratory analysis

Glucagon-like-peptide-1 (GLP-1) will be measured with a commercially available ELISA kit (Millipore Corporation, Billerica, Massachusetts, USA). This kit is for non-radioactive quantification of GLP-1 (7-36) in serum and EDTA-plasma samples; it is high specific and does not detect other forms of GLP-1. The intra- and interassay coefficient of variation is below 9.0% and 13.0%, respectively.

Peptide tyrosine tyrosine (PYY) will be measured with a commercially available radioimmunoassay kit (Millipore Corporation, Billerica, Massachusetts, USA). The anti-PYY-antibody used in this kit is raised in guinea pigs and displays 100% cross-reactivity with human PYY1-36 and human PYY3-36, but no cross-reactivity with human pancreatic polypeptide, NPY and unrelated peptides such as leptin and ghrelin. The intra- and inter-assay coefficient of variation for this assay is below 9.4 % and 8.5 %, respectively.

Cholecystokinin (CCK) will be measured with a commercially available radioimmunoassay kit (Euro-Diagnostica, Malmö, Sweden). This kit is for assay of CCK in plasma. The intra- and interassay coefficient of variation for this assay is below 5.5% and 13.7%, respectively.

Total ghrelin will be measured with a commercially available radioimmunoassay kit (Millipore Corporation, Billerica, Massachusetts, USA). This kit is for quantitative determination of total ghrelin in serum, plasma or tissue culture media. It is highly specific for ghrelin and shows no cross-reactivity with other hormones, like glucagon, leptin or insulin. The intra- and inter-assay coefficient of variation for this assay is below 10.0 % and 14.7 %, respectively.

Insulin will be measured with a commercially available ELISA kits (Abnova, Taipei City, Taiwan). This kit is for quantitative determination of insulin in human plasma (EDTA). It is highly specific for insulin and shows no cross-reactivity with other peptides, e.g. c-peptide or glucagon. The intra- and inter-assay coefficient of variation is below 8.1% and 8.5%, respectively.

Plasma glucose concentration will be measured by a commercially available glucoseoxidase-method (Bayer Consumer Care AG, Basel, Switzerland). This method is highly specific for measurement of glucose in serum or plasma.

Statistical analysis

Descriptive statistics will be used for demographic variables such as age, weight, height, and BMI. Measures of task performance, appetite ratings, physiological data, hormone levels and gastric emptying rates will be analysed using repeated measures ANOVAs. When significant differences will be found, the Tukey's test for pairwise comparisons will be applied. However, if due to the limited sample size for the behavioral part precludes a parametric approach, a nonparametric statistical test procedure will be applied (Kolmogorov–Smirnov one sample test). Individual hormone concentrations versus time data will be used to obtain plasma GLP-1, PYY, CCK, ghrelin, insulin, glucose and amino acid metrics, including maximum/minimum plasma concentrations (Cmax/Cmin), the time of maximal/minimal peptide occurrence (Tmax/Tmin) and the area under the plasma concentration-time profile (AUC). Pharmacokinetic parameters (Cmax, tmax, AUC, etc.) will be obtained using PK Functions for Excel®. AUC values will be calculated by the trapezoidal rule. Differences between treatments will be assessed using either the non-parametric Friedman-test (in case of significant differences followed by pairwise comparison using the Wilcoxon signed ranks test and Bonferroni‘s correction to account for multiple of comparisons); or the General linear model procedure of repeated-measures ANOVA using simple contrast and Bonferroni correction of P values for multiplicity of comparison. VAS will be analysed by calculating AUC and return to baseline values (interception with y-axis using linear interpolation). Differences will be assessed using the non-parametric Friedman-test or the General linear model procedure of repeated-measures ANOVA. Transformations will be performed before analysis, if response variables are non-normally distributed. All statistical analysis will be done

using SPSS for windows software (version 19.0; SPSS Inc, Chicago, Illinois). Values will be reported as mean ± SEM. Differences will be considered to be significant at $P \leq 0.05$. Based on past experience, a sample size of 12-16 subjects per protocol should allow detecting differences of 15 to 25% in the means of our endpoint parameters with a statistical power of 80%.

References

1. Fromentin G, Darcel N, Chaumontet C, Marsset-Baglieri A, Nadkarni N, Tome D. Peripheral and central mechanisms involved in the control of food intake by dietary amino acids and proteins. Nutrition research reviews. 2012;25(1):29-39. Epub 2012/05/31.
2. Bowen J, Noakes M, Clifton PM. Appetite regulatory hormone responses to various dietary proteins differ by body mass index status despite similar reductions in ad libitum energy intake. J Clin Endocrinol Metab. 2006;91(8):2913-9. Epub 2006/06/01.
3. Tappy L. Thermic effect of food and sympathetic nervous system activity in humans. Reproduction, nutrition, development. 1996;36(4):391-7. Epub 1996/01/01.
4. Mithieux G. A novel function of intestinal gluconeogenesis: central signaling in glucose and energy homeostasis. Nutrition. 2009;25(9):881-4. Epub 2009/08/04.
5. Potier M, Darcel N, Tome D. Protein, amino acids and the control of food intake. Current opinion in clinical nutrition and metabolic care. 2009;12(1):54-8. Epub 2008/12/06.
6. Bowen J, Noakes M, Trenerry C, Clifton PM. Energy intake, ghrelin, and cholecystokinin after different carbohydrate and protein preloads in overweight men. J Clin Endocrinol Metab. 2006;91(4):1477-83. Epub 2006/01/26.
7. Lejeune MP, Westerterp KR, Adam TC, Luscombe-Marsh ND, Westerterp-Plantenga MS. Ghrelin and glucagon-like peptide 1 concentrations, 24-h satiety, and energy and substrate metabolism during a high-protein diet and measured in a respiration chamber. The American journal of clinical nutrition. 2006;83(1):89-94. Epub 2006/01/10.
8. Johnson J, Vickers Z. Effects of flavor and macronutrient composition of food servings on liking, hunger and subsequent intake. Appetite. 1993;21(1):25-39. Epub 1993/08/01.
9. Batterham RL, Heffron H, Kapoor S, Chivers JE, Chandarana K, Herzog H, et al. Critical role for peptide YY in protein-mediated satiation and body-weight regulation. Cell Metab. 2006;4(3):223-33. Epub 2006/09/05.
10. Thimister PW, Hopman WP, Sloots CE, Rosenbusch G, Willems HL, Trijbels FJ, et al. Role of intraduodenal proteases in plasma cholecystokinin and pancreaticobiliary responses to protein and amino acids. Gastroenterology. 1996;110(2):567-75. Epub 1996/02/01.
11. Mellinkoff SM, Frankland M, Boyle D, Greipel M. Relationship between serum amino acid concentration and fluctuations in appetite. Journal of applied physiology. 1956;8(5):535-8. Epub 1956/03/01.
12. Gannon MC, Nuttall FQ. Amino acid ingestion and glucose metabolism--a review. IUBMB life. 2010;62(9):660-8. Epub 2010/10/01.

13. Greenfield JR, Farooqi IS, Keogh JM, Henning E, Habib AM, Blackwood A, et al. Oral glutamine increases circulating glucagon-like peptide 1, glucagon, and insulin concentrations in lean, obese, and type 2 diabetic subjects. The American journal of clinical nutrition. 2009;89(1):106-13. Epub 2008/12/06.
14. Liddle RA. Regulation of cholecystokinin secretion in humans. Journal of gastroenterology. 2000;35(3):181-7. Epub 2001/02/07.
15. Ballinger AB, Clark ML. L-phenylalanine releases cholecystokinin (CCK) and is associated with reduced food intake in humans: evidence for a physiological role of CCK in control of eating. Metabolism: clinical and experimental. 1994;43(6):735-8. Epub 1994/06/01.
16. Colombel JF, Sutton A, Chayvialle JA, Modigliani R. Cholecystokinin release and biliopancreatic secretion in response to selective perfusion of the duodenal loop with aminoacids in man. Gut. 1988;29(9):1158-66. Epub 1988/09/01.
17. Thomas FB, Sinar D, Mazzaferri EL, Cataland S, Mekhjian HS, Caldwell JH, et al. Selective release of gastric inhibitory polypeptide by intraduodenal amino acid perfusion in man. Gastroenterology. 1978;74(6):1261-5. Epub 1978/06/01.
18. Wang Y, Chandra R, Samsa LA, Gooch B, Fee BE, Cook JM, et al. Amino acids stimulate cholecystokinin release through the Ca2+-sensing receptor. American journal of physiology Gastrointestinal and liver physiology. 2011;300(4):G528-37. Epub 2010/12/25.
19. Liou AP, Sei Y, Zhao X, Feng J, Lu X, Thomas C, et al. The extracellular calcium-sensing receptor is required for cholecystokinin secretion in response to L-phenylalanine in acutely isolated intestinal I cells. American journal of physiology Gastrointestinal and liver physiology. 2011;300(4):G538-46. Epub 2011/01/22.
20. Blundell J. Pharmacological approaches to appetite suppression. Trends in pharmacological sciences. 1991;12(4):147-57. Epub 1991/04/01.
21. Lieverse RJ, Jansen JB, Masclee AM, Lamers CB. Satiety effects of cholecystokinin in humans. Gastroenterology. 1994;106(6):1451-4. Epub 1994/06/01.
22. Flint A, Raben A, Blundell JE, Astrup A. Reproducibility, power and validity of visual analogue scales in assessment of appetite sensations in single test meal studies. International journal of obesity and related metabolic disorders : journal of the International Association for the Study of Obesity. 2000;24(1):38-48.
23. Steinert RE, Meyer-Gerspach AC, Beglinger C. The role of the stomach in the control of appetite and the secretion of satiation peptides. Am J Physiol Endocrinol Metab. 2012;302(6):E666-73. Epub 2012/01/05.
24. Gerspach AC, Steinert RE, Schonenberger L, Graber-Maier A, Beglinger C. The role of the gut sweet taste receptor in regulating GLP-1, PYY, and CCK release in humans. Am J Physiol Endocrinol Metab. 2011;301(2):E317-25. Epub 2011/05/05.
25. Blundell J, de Graaf C, Hulshof T, Jebb S, Livingstone B, Lluch A, et al. Appetite control: methodological aspects of the evaluation of foods. Obesity reviews : an official journal of the International Association for the Study of Obesity. 2010;11(3):251-70. Epub 2010/02/04.
26. Braden B, Adams S, Duan LP, Orth KH, Maul FD, Lembcke B, et al. The [13C]acetate breath test accurately reflects gastric emptying of liquids in both liquid and semisolid test meals. Gastroenterology. 1995;108(4):1048-55. Epub 1995/04/01.

27. Ghoos YF, Maes BD, Geypens BJ, Mys G, Hiele MI, Rutgeerts PJ, et al. Measurement of gastric emptying rate of solids by means of a carbon-labeled octanoic acid breath test. Gastroenterology. 1993;104(6):1640-7. Epub 1993/06/01.
28. Veereman-Wauters G, Ghoos Y, van der Schoor S, Maes B, Hebbalkar N, Devlieger H, et al. The 13C-octanoic acid breath test: a noninvasive technique to assess gastric emptying in preterm infants. Journal of pediatric gastroenterology and nutrition. 1996;23(2):111-7. Epub 1996/08/01.
